# Domestication as a Novel Approach for Improving the Cultivation of Calanoid Copepods: A Case Study with *Parvocalanus crassirostris*

**DOI:** 10.1371/journal.pone.0133269

**Published:** 2015-07-17

**Authors:** Fahad Alajmi, Chaoshu Zeng, Dean R. Jerry

**Affiliations:** Centre for Sustainable Tropical Fisheries and Aquaculture and School of Marine and Tropical Biology, James Cook University, Townsville, Queensland 4811, Australia; Stazione Zoologica Anton Dohrn, ITALY

## Abstract

Calanoid copepods are an important food source for most fish larvae. Their role as a natural prey item means that it is important to develop culture technology for copepods to meet the requirements of larvae culture in aquaculture hatcheries. Copepods have been cultured successfully for some time; however, the implications of long-term cultivation or domestication on copepod life history traits have not yet been assessed. Therefore, the aim of this study was to determine if the domesticated and wild populations of *Parvocalanus crassirostris* are phenotypically or physiologically different. Wild-caught *P*. *crassirostris* were compared to a long-held domesticated strain (>2 years) for size of developmental stage, fecundity and lifespan of adult females, culture density, ingestion rates, faecal pellet production and fatty acid profiles. The domesticated strain was significantly different from the wild strain in size (eggs, nauplii, copepodites and adults were larger in the domesticated strain), egg production (112.3 ± 1.8 eggs female^-1^ vs. 64.6 ± 3.3 eggs female^-1^) and adult female lifespan (8.8 ± 0.1 days vs., 7.5 ± 0.1 days). At 1, 3 and 5 adults mL^-1^, the domesticated strain performed significantly better than the wild strain in egg production (4189.8 ± 61.2, 11224.0 ± 71.7 and 21860.6 ± 103.6 eggs vs. 1319.5 ± 54.3, 2374.5 ± 80.9 and 4933.8 ± 269.5 eggs, respectively) and mean daily mortality rate (5.6% across all densities vs. 22.9 ± 1.6, 29.8 ± 1.2 and 31.3 ± 1.3%, respectively). The domesticated strain had significantly higher ingestion rates than the wild stain (888.4 ± 9.9 ng C l^-1^ and 775.3 ± 11.2 ng C l^-1^, respectively), while faecal pellet production was not significantly different between strains. Fatty acid profiles indicated higher levels (as % of total fatty acid) of long-chain polyunsaturated fatty acids in the domesticated strain (36.2±0.4%) than the wild strain (16.1±0.1%). Overall, this study found that the reproductive capacity and tolerance to the culture environment of the calanoid *P*. *crassirostris* have improved significantly due to domestication.

## Introduction

For the past three decades, there has been continuing interest in the development of mass culture techniques for copepods to be used as live food in aquaculture [1–4]. Copepods are the most common metazoans in the marine environment [5], with approximately 11,500 described species [6, 7]. Unlike traditional live feeds such as rotifers, copepod nutritional profiles are rich with essential fatty acids, free amino acids and other essential micronutrients [8, 9]. Moreover, the small size of copepod nauplii is vital for the first larval feeding of various fish species with small mouth gape sizes, such as groupers and snappers [4, 10]. Copepods also stimulate strong feeding responses in fish larvae through their distinctive swimming patterns [11–13]. Nevertheless, utilisation of copepods in aquaculture hatcheries is still sporadic, which is largely attributed to difficulties in their culture and, in particular, their low culture productivity [14].

Calanoid copepods dominate the water column in marine environments and are generally planktonic through all of their life stages [5]; consequently, calanoid copepods are considered to be the best candidates for first feeding of fish larvae in aquaculture [15, 16]. Previous research has demonstrated many difficulties in mass culturing calanoid copepods due to their low tolerance to changes in water quality [4] and low culture productivity compared to other taxonomic groups [17]. Recently, several calanoid species have been identified as great candidates for aquaculture, including the calanoids *Acartia tons*, *Acartia sinjiensis* and *Parvocalanus crassirostris*, which have been cultured over multiple generations in laboratories and aquaculture facilities [18–21]. Despite the increased use of domesticated copepods, the implications of long-term cultivation or domestication on copepod life history traits has not been quantified to date.

Generally, domestication is characterized by genetic changes in behaviour, morphology, or physiology caused by cultivation under artificial conditions [22]. The benefits of domestication have been demonstrated for many aquaculture species, with improved productivity and tolerance to culture conditions resulting simply from culturing a species for several generations without direct selection [23, 24]. However, domestication could also result in loss of fitness, particularly if pedigrees are not adequately kept and inbreeding occurs. Furthermore, the effects of domestication are likely to accumulate over generations [25].

In comparison, copepod generation times are extremely short compared to most other cultured aquatic species (i.e., weeks). These multiple generations within a short time could result in either positive or negative phenotypic responses due to changes in environmental conditions. For example, inconsistent behaviour and reproductive capacity was found between laboratory-cultured and wild *Acartia tonsa*, with more eggs produced by laboratory-cultured copepods [26]. Furthermore, significant differences in fatty acid profiles have been reported between captive and wild copepods [8, 27]. These changes in vital culture characteristics warrant further investigation into the effects of domestication that might have occurred over multiple generations.

The calanoid copepod *Parvocalanus crassirostris* has been isolated from the wild and cultivated as a closed population at James Cook University, Queensland, for over 2 years. *P*. *crassirostris* is a small copepod found in tropical and subtropical waters globally [28–30]. *P*. *crassirostris* attains higher culture densities and productivity than previously investigated calanoid species [4, 18–20, 31, 32]. These positive attributes indicate a potential for *P*. *crassirostris* to be used successfully in an aquaculture hatchery as live food for fish larvae. The short life cycle of *P*. *crassirostris* (≈18 days) theoretically means that more than 80 generations have passed since the James Cook University domesticated strain was collected from the wild. Because there was no intentional selection on the base population for any trait, this population offers a unique opportunity to evaluate the effects of domestication on a range of biological traits in comparison to wild copepod populations. To date, there is no information on the potential effects of domestication of copepods on traits relevant to culture productivity and utilization in aquaculture (i.e., reproductive capacity). Therefore, the aim of this experiment was to investigate the effects of domestication after multiple generations of controlled cultivation to determine if the domesticated and wild populations of *P*. *crassirostris* are phenotypically or physiologically different.

## Materials and Methods

### Copepod cultures

*Parvocalanus crassirostris* were initially collected from plankton tows in coastal waters off of Townsville, Northern Queensland, Australia (Lat. 19° 15' 3" S, Long. 146° 49' 17" E), in August 2011. The field location was not privately-owned or protected in any way and our study did not involve endangered or protected species. No specific permits were required for collecting samples in that location. The copepods were cultured at James Cook University from an initial founder population of 300 to 400 adults and used in the present experiments as a domesticated strain. To ensure the same genetic stock was sampled as the domesticated strain, wild *P*. *crassirostris* were collected from the original location of the earlier collection in September 2013. Pure copepod cultures were maintained in 20-L polycarbonate carboys filled with 1 μm filtered seawater (36±1) with gentle aeration. The culture temperature was kept at 26 ±1°C, and a photoperiod of 16:8 h light/dark cycle was maintained. The propensity of the culture salinity to increase due to evaporation was adjusted by adding dechlorinated distilled water if needed. Experiments did not commence until wild *P*. *crassirostris* completed at least one generation of breeding post-collection to ensure that the differences between the two stocks were not simply due to a lack of acclimatization to the experimental environment and/or stress.

Partial water changes were performed weekly with the removal of detritus by siphoning the bottom of the carboys. To ensure that adult copepods were not siphoned, siphoning was performed with a 100-μm mesh attached to the end of the siphoning tube. The siphoned water was collected and sieved through a 25-μm mesh, and the eggs and nauplii retained on the mesh were re-suspended in a 1-L beaker. The beaker was left to settle for 2 days to allow eggs to hatch before the water was gently poured onto a 25-μm mesh to collect nauplii to return them to the stock culture. Debris left at the bottom of the beaker was discarded.

Copepods were fed the Tahitian strain of *Isochrysis* sp. every second day at 1000 μg C l^-1^ based on [31]. When the culture density in a carboy reached 4–5 individuals ml^-1^, the carboy content was divided to initiate a new culture, and through this procedure, a continuously growing stock was maintained.

### Microalgae culture

The Tahitian strain of *Isochrysis* sp. (T-Iso) used in this study was originally obtained from the microalgae supply service at the Commonwealth Scientific and Industrial Research Organisation (CSIRO, Australia). T-Iso was cultured in a temperature-controlled room using 20-L polycarbonate carboys filled with 1-μm filtered, autoclaved and UV-irradiated seawater at a salinity of 35 ppt. T-Iso was cultured using f/2 medium (Guillard and Ryther, 1962). All cultures were maintained at a temperature of 25 ±1°C with vigorous aeration (0.2 μm filtered air). The photoperiod was set to a light/dark cycle of 16:8 h with a light intensity of approximately 65 μmol m^−1^ s^−1^ (fluorescent). The algal cultures were in their exponential growth phase when used to feed the copepods.

### Life history traits

The size of developmental stages, fecundity and lifespan of adult females were evaluated to determine the response to domestication. One hundred randomly collected individuals of each stage (eggs to adults) from each population were measured. Size measurements were made using a Leica DM LB microscope and an Olympus DP25 camera. Photos were analysed with Olympus DP2-BSW software. Fecundity and lifespan were evaluated using a design adapted from [33]. Pre-matured females were collected from each population at the final copepodite stage V (CV) and then individually isolated in petri dishes to be observed until sexual maturity was reached. Maturation was confirmed by the moult of the CV copepodites (Lawson and Grice, 1973). Each mature adult female was paired with an adult male (replicates, *n* = 10). Each pair was then cultured in a 200-mL vessel submerged in a beaker. A 74-μm mesh was attached to the bottom to ensure that the adults would remain inside the vessel while eggs would pass through and accumulate at the bottom of the beaker. The eggs that accumulated at the bottom of the beakers were counted following daily water exchanges with a Bogorov counting chamber and a dissecting stereo-microscope (Nikon SMZ645). Similarly, the lifespan of adult females was evaluated. The 200-mL culture vessels were stocked with 10 pre-matured adults (*n* = 10) from each population and monitored every day for mortality. Dead copepods were removed daily. The experiment stopped when all copepods died.

### Culture density

Three stocking density treatments of 1, 3 and 5 adults mL^-1^ were evaluated (*n* = 10) following the methods described in [19]. Stage V copepodites (CV) were randomly collected from stock cultures of the wild and domesticated populations and then cultured in 200-mL vessels at designated densities. Sex ratio of all density treatments was not manipulated and therefore was within the normal range of the species (85%- 90% females). The experiment started 24 h after stocking CV individuals to allow time for their development into adults [31]. Mean daily egg production was evaluated over 7 days of culture, while the mean daily mortality rate of adults was calculated over 5 days of culture using the following formula:

$$\text{Daily mortality \% }=\left[\frac{\text{No. of dead copepods found on a particular day}}{\text{Total surviving adults of the previous day}}\right]\text{ x100}$$

### Ingestion rate and faecal pellet production

To evaluate the ingestion rate, 30 recently matured females (*n* = 10) were cultured in 75-mL containers filled with fresh seawater (36±1) and T-Iso at 1000 μg C l^-1^. Culture containers were rotated for 24 h on a plankton wheel at 1 rpm to maintain a constant suspension. Five control replicates without copepods were additionally maintained. After 24 h, all individuals were removed from the containers and mortality was calculated. Initial (C_0_) and final (C_t_) algae concentrations were calculated and the ingestion rate (I) was evaluated according to [34] as follows:

$$F=\frac{V\left(\frac{\text{ln}C_{0}-\text{ln}C_{t}}{t}\right)+k}{N}$$

I = F X C_0_

F = clearance rate (μg C l^-1^ female^-1^ h^-1^); V = container volume (mL); N = number of individuals; C_0_ = initial algae concentration (μg C l^-1^); C_t_ = final algae concentration (μg C l^-1^); t = time (h); k = cell growth coefficient (h^-1^).

Faecal pellet production was measured using similar methods described for the collection of eggs (described above). Recently matured females from wild and domesticated populations were individually cultured for the duration of their lifespan, and their daily faecal pellet production was counted.

### Fatty acid analysis

To obtain an adequate sample for fatty acid analysis, *P*. *crassirostris* were cultured in 250-L tanks fed the designated diet treatments under similar conditions described for main stock cultures (described above). *P*. *crassirostris* were harvested after 7 days of culture by filtering the content of the culture tanks through a 150-μm mesh followed by a 25-μm mesh. This process ensured the collection of second generation eggs, nauplii and early copepodites (I-III). The selected size fraction represented the dominant component of the cultured population. The collected copepod samples were then flushed three times in 10‰ saltwater to remove salt and transferred to 2-mL vials for freeze drying.

Fatty acid methyl esters (FAMEs) were prepared by transesterification using 14% BF, (boron trifluoride/methanol, w/w), following the method of [35]. FAMEs were quantified by an Agilent 6890 gas chromatograph (Agilent Technologies, Santa Clara, CA) coupled to a flame ionization detector, with an Omegawax 320 fused silica capillary column (30 m x 0.32 mm; Supelco, Bellefonte, PA). The injector and detector temperature was maintained at 260°C. The column temperature was initially set to 60°C, then increased at a rate of 50°C min^-1^ to 170°C, followed by an increase at a rate of 2°C min^-1^ to 180°C, and was then held for 2 min. The temperature was further increased at the same rate of 2°C min^-1^ to 230°C and held for 1 min before another increase at a rate of 1°C min^-1^ to the final temperature of 240°C, until all FAMEs had been eluted. The total time was 46.2 min. The carrier gas was helium with a flow velocity of 25 cm s^-1^. Peaks were identified by comparing retention times with known standards (Nu-Chek-Prep Inc, Elysian, MN). Fatty acid contents were expressed as a percentage of a particular fatty acid to the total fatty acids (%).

### Statistical analysis

Data are presented as the mean ± standard error (SE), except for fatty acid composition data (mean ± standard deviation). All data collected were tested for normality and homogeneity of variance prior to mean comparison procedures, and data expressed in percentages were arcsin-transformed prior to analysis. Size measurements, ingestion rates and fatty acid composition data were compared using one-way ANOVA, while egg and faecal pellet production data were analysed with repeated-measures ANOVA. When significant differences (p <0.05) were found, Tukey’s multiple comparison test was performed to determine specific differences among treatments. All statistical analyses were conducted using Sigmaplot, version 11.

## Results

### Life history traits

Table 1 summarizes the size measurements of domesticated and wild developmental stages. Generally, size over all developmental stages was significantly different between strains (p <0.001), with domesticated individuals being significantly larger than the wild strain.

**Table 1:** Mean length (μm) of developmental stages of domesticated and wild *P*. *crassirostris*.

	Strain	
Stage	Domesticated	Wild
Egg	71.0 ± 0.4^a^	66.9 ± 0.4^b^
Nauplii I	80. ± 0.6^a^	76.8 ± 0.5^b^
Nauplii II	87.4 ± 0.4^a^	83.5 ± 0.5^b^
Nauplii III	112.1 ± 0.4^a^	106.3 ± 0.4^b^
Nauplii IV	134.8 ± 0.8^a^	126.1 ± 0.9^b^
Nauplii V	155.6 ± 0.8^a^	150.0 ± 0.9^b^
Nauplii VI	172.7 ± 0.7^a^	165.9 ± 0.8^b^
Copepodite I	195.4 ± 0.6^a^	185.1 ± 0.6^b^
Copepodite II	257.1 ± 1.1^a^	245.0 ± 1.2^b^
Copepodite III	299.7 ± 1.7^a^	290.2 ± 1.7^b^
Copepodite IV	338.9 ± 1.1^a^	332.0 ± 1.2^b^
Copepodite V	389.8 ± 1.1^a^	381.2 ± 1.3^b^
Adult male	384.1 ± 0.8^a^	345.0 ± 0.9^b^
Adult female	444.7 ± 2.9^a^	432.6 ± 2.5^b^

Lengths of copepodites and adults were measured as the prosome length only. Data are presented as mean±S.E (*n* = 100).

^ab^Different letter superscripts in the same row indicate statistical difference (p < 0.05).

**Fig 1:**
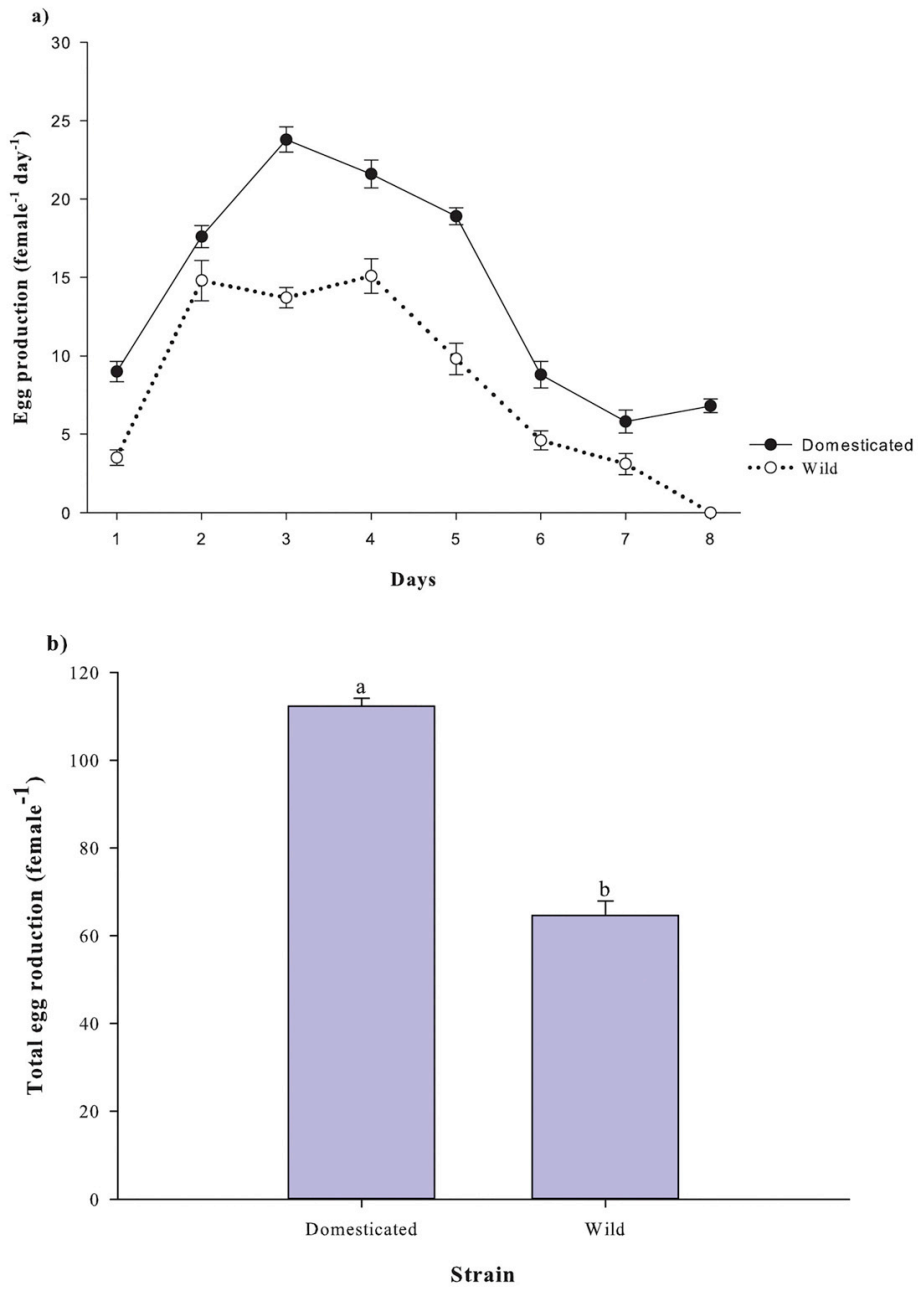
Egg production of *P*. *crassirostris* (female^-1^) strains (*n* = 10). a) Mean daily egg production female^-1^ over 8 days; b) mean total egg production female^-1^ over 8 days. Data are presented as the mean ± standard error. The different letters on the tops of the bars indicate significant differences (p <0.001).

Within each strain, *P*. *crassirostris* adult females displayed a similar daily egg production pattern over their lifespan. On any given day, the domesticated female's daily egg production was significantly higher than the wild strain (p <0.001; Fig 1a). The domesticated females continued to produce eggs on day 8, whereas all females from the wild strain ceased egg production on day 7. The mean total egg production over a female’s lifespan for the domesticated strain was significantly higher compared with the wild strain at 112.3 ± 1.8 vs. 64.6 ± 3.3 eggs female^-1^, respectively (p <0.001; Fig 1b). Thus, when compared to the wild strain, the domesticated copepods produced 73.8% more eggs per female.

**Fig 2:**
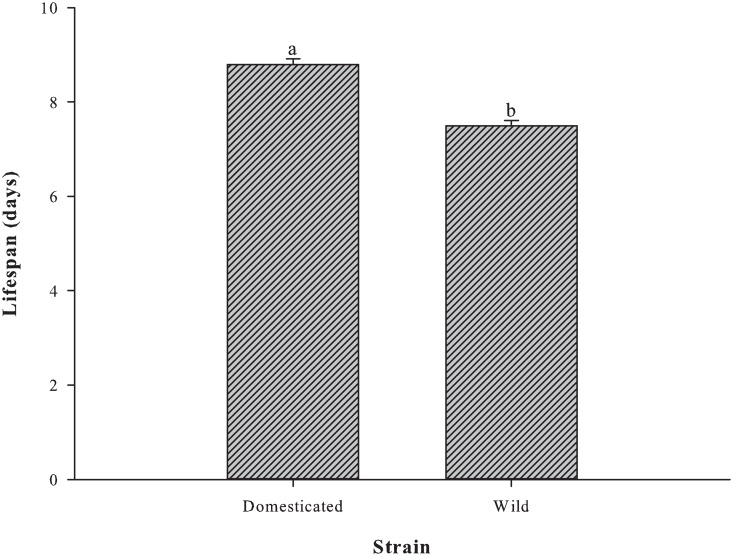
Adult female lifespan of *P*. *crassirostris* strains (*n* = 10). The different letters on the tops of the bars indicate significant differences (p <0.001).

The mean female lifespans of the *P*. *crassirostris* strains were significantly different (p <0.001; Fig 2). Domesticated females had a longer lifespan (8.8 ± 0.1 days), while wild females lived a day less (7.5 ± 0.1 days).

### Culture density

**Fig 3:**
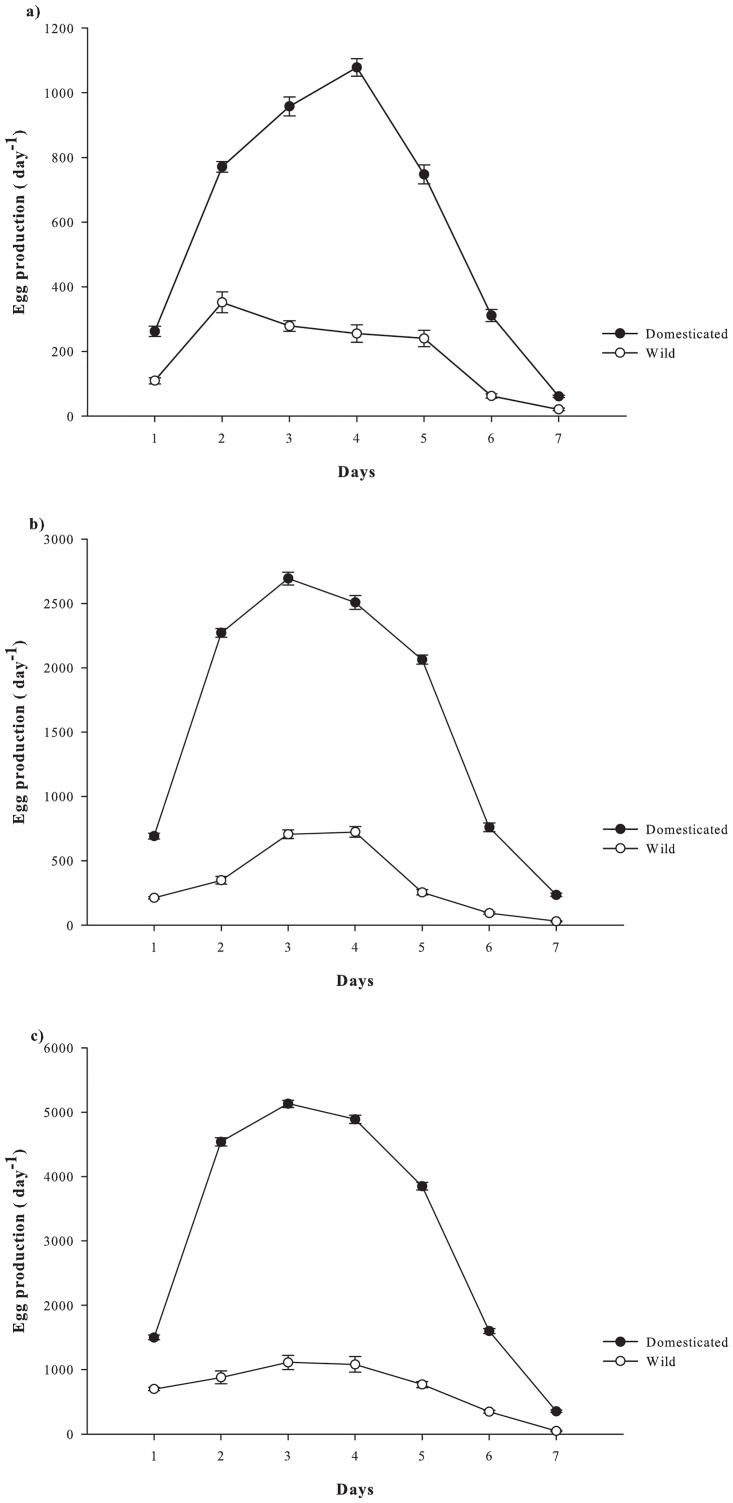
Mean daily egg production of domesticated and wild *P*. *crassirostris* strains cultured at initial densities of (a) 1, (b) 2 and (c) 3 mL^-1^ (*n* = 10). Data are presented as mean ± standard error.

Fig 3 shows the mean daily egg production of *P*. *crassirostris* over 7 days at different initial stocking densities. The differences in mean daily egg production were highly significant between strains at different density treatments (p <0.001). Two very different patterns were demonstrated between the strains; the first pattern was characterized by a low mean egg production on the first day but increased sharply on day 2, with a peak production between days 2 and 5. A steep decrease followed on days 6 and 7, although egg production was still found on day 7. This pattern was shown by the domesticated strain at stocking densities of 1, 3 and 5 adults mL^-1^. The second pattern was characterized by steady and substantially lower mean egg production from days 2 to 5, followed by a significant drop in mean egg production on day 6 and the termination of all egg production after day 6. This pattern was shown by the wild strain at stocking densities of 1, 3 and 5 adults mL^-1^.

**Fig 4:**
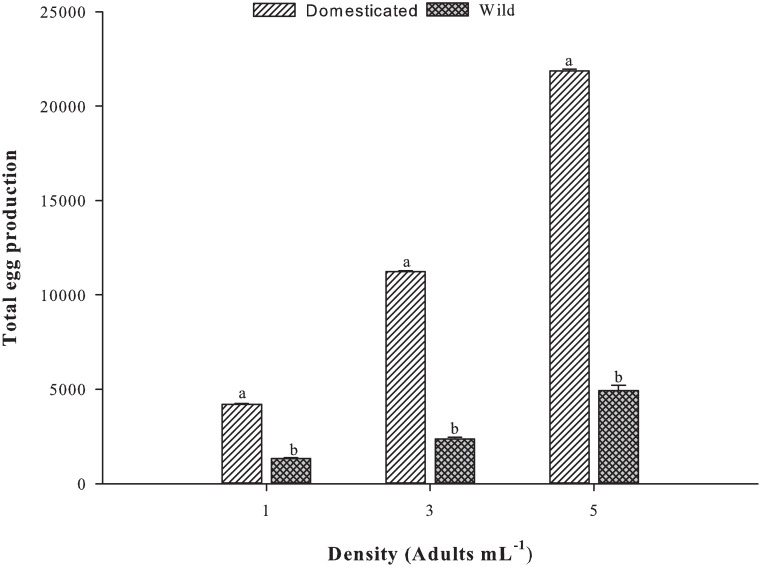
Mean total egg production of *P*. *crassirostris* strains under different initial adult stocking densities (*n* = 10). The different letters on the tops of the bars indicate significant differences (p <0.001).

Mean total egg production over 7 days was also significantly different across different stocking densities (p >0.001; Fig 4). The domesticated females at all stocking densities produced significantly more total eggs than the wild strain (p <0.001). The domesticated strain produced 4189.8 ± 61.2, 11224.0 ± 71.7 and 21860.6 ± 103.6 eggs at 1, 3 and 5 adults mL^-1^, respectively, while the wild strain produced 1319.5 ± 54.3, 2374.5 ± 80.9 and 4933.8 ± 269.5 eggs at 1, 3 and 5 adults mL^-1^, respectively.

**Fig 5:**
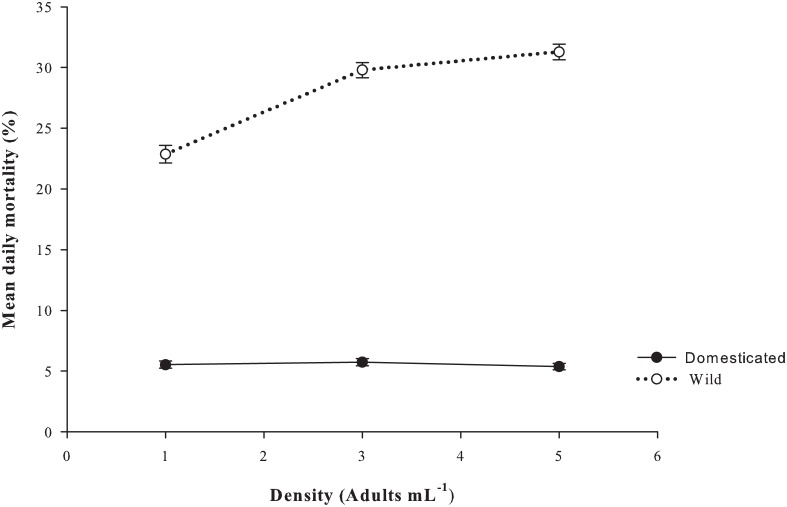
Mean daily mortality rate (%) of *P*. *crassirostris* strains under different initial adult stocking densities (*n* = 10). Data are presented as the mean ± standard error.

The mean daily mortality rates were significantly different between the two strains (p <0.001; Fig 5). While the mean daily mortality rate of the domesticated strain remained very low for the 1, 3 and 5 adults mL^-1^ treatments (5.6 ± 0.3, 5.6 ± 0.1 and 5.5 ± 0.3%, respectively) and no significant differences were detected between all densities (p >0.05), the wild strain had a significantly higher mean daily mortality rate across all densities (p <0.001). The wild strain’s mean daily mortality rates were 22.9 ± 1.6, 29.8 ± 1.2 and 31.3 ± 1.3% at 1, 3 and 5 adults mL^-1^, respectively, with no differences between treatments (p >0.05).

### Ingestion rate and faecal pellet production

**Fig 6:**
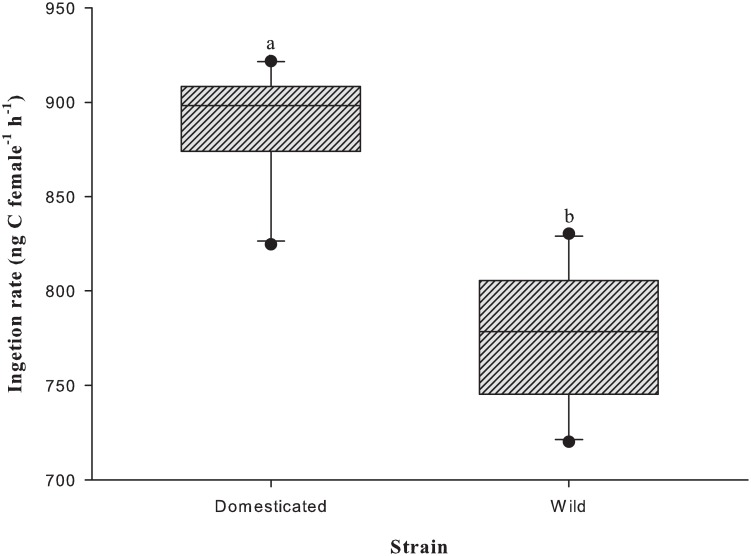
Mean ingestion rate (ng C female^-1^ h^-1^) of *P*. *crassirostris* strains (*n* = 10) at an average food concentration of 1000 μg C l^-1^. The different letters on the tops of the boxes indicate significant differences (p <0.01).

Ingestion rates obtained at an average food concentration of 1000 μg C l^-1^ were significantly different between the two strains (p <0.001; Fig 6). The domesticated strain had a significantly higher ingestion rate at 888.4 ± 9.9 ng C l^-1^ compared to 775.3 ± 11.2 ng C l^-1^ for the wild strain (p <0.001). The domesticated strain grazed more efficiently compared to the wild strain, as depicted by a higher clearance rate of 0.9 ± 0.0 mL female^-1^ h^-1^ compared to 0.8 ± 0.0 mL female^-1^ h^-1^ (p <0.001).

**Fig 7:**
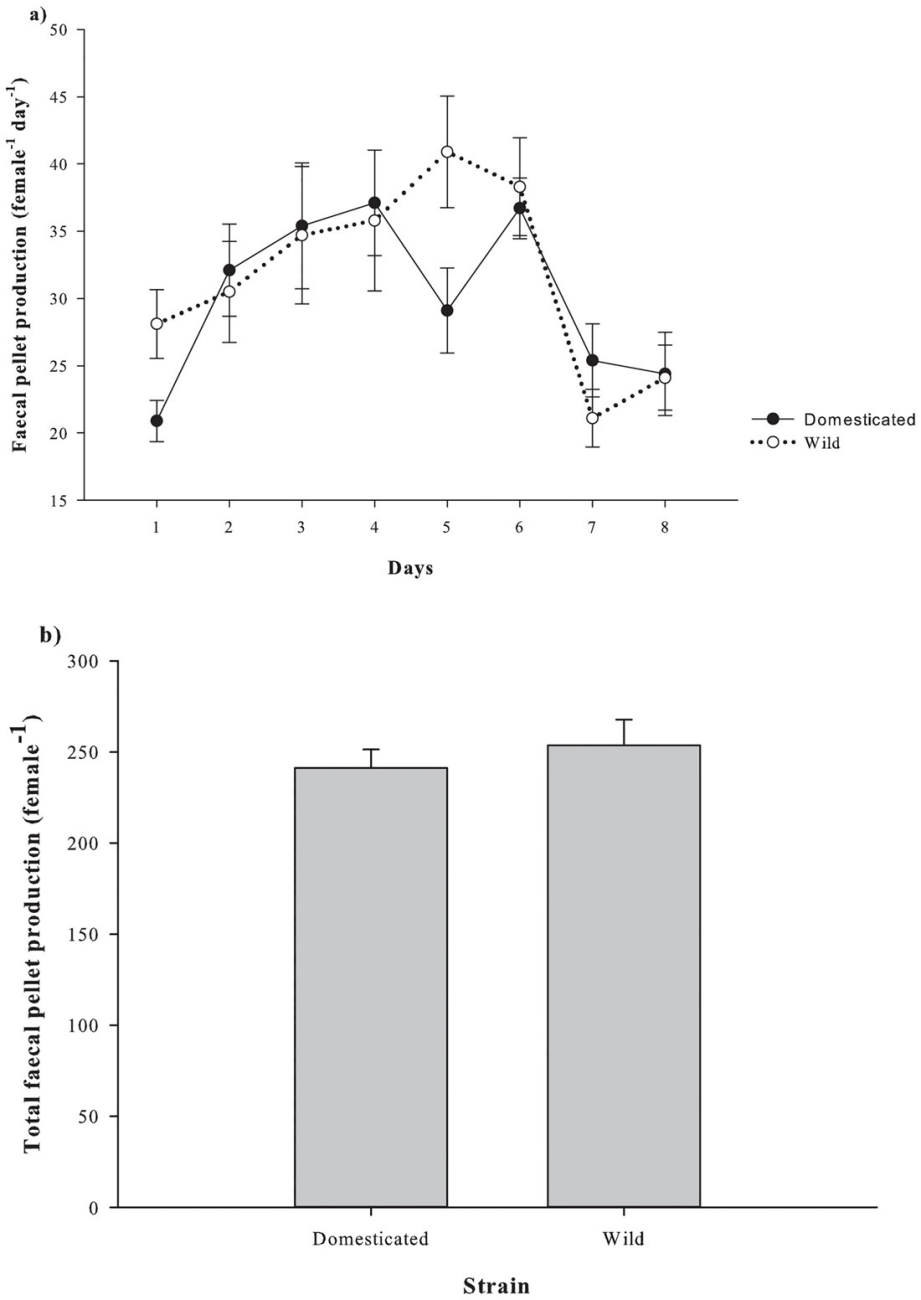
Faecal pellet production (female^-1^ day^-1^) of *P*. *crassirostris* strains (*n* = 10). a) Mean daily faecal pellet production female^-1^ over 8 days; b) mean total faecal pellet production female^-1^ over 8 days. Data are presented as the mean ± standard error.

Mean daily faecal pellet production (female^-1^ day^-1^) was not significantly different between strains (p >0.05; Fig 7a), at 30.1 ± 1.2 and 31.7 ± 1.7 female^-1^ day^-1^ for the domesticated and wild strain, respectively. Furthermore, the difference in the total faecal pellet production (female^-1^) between strains was not statistically significant (p >0.05; Fig 7b). The mean total faecal pellets produced were 241.1 ± 10.2 and 253.5 ± 14.2 female^-1^ for the domesticated and wild strains.

### Fatty acid analysis

Table 2 summarizes the fatty acid composition of *P*. *crassirostris* strains. The domesticated copepods had significantly higher levels of C18:2ω6 (4.7±0.1%) and C18:3ω3 (3.3±0.1%) than the wild copepods (3.2±0.0 and 2.3±0.1%, respectively), while C20:4ω6 levels were not significantly different between strains. Domesticated copepods had significantly higher levels of EPA and DHA (5.4±0.1 and 22.3±0.3%, respectively) than the wild copepods (2.7±0.1 and 6.1±0.1%, respectively). LC-PUFA levels were significantly higher in the domesticated copepods (36.2±0.4%) compared to wild copepods (16.1±0.1%).

**Table 2:** Fatty acid composition of domesticated and wild cultured strains of *P*. *crassirostris* fed *Isochrysis* sp. (mean % of total fatty acids ± SD).

	Strain		
Fatty acids	Domesticate (*n* = 3)	Wild (*n* = 3)	ANOVA (p value)
C14:0	8.8±0.1	6.7±0.1	.000
C15:0	0.6±0.1	0.7±0.0	.212
C16:0	16.5±0.1	20.1±0.1	.000
C18:0	5.2±0.1	7.8±0.1	.000
C22:0	0.4±0.0	0.3±0.0	.001
C23:0	0.5±0.0	1.1±0.0	.000
C24:0	0.4±0.0	0.7±0.0	.000
C14:1ω7	1.0±0.0	0.5±0.0	.000
C16:1ω7	4.5±0.1	4.2±0.1	.025
C16:1ω5	0.5±0.0	0.4±0.0	.011
C18:1ω9	7.1±0.1	11.3±0.1	.000
C18:1ω7	3.5±0.1	3.1±0.1	.007
C20:1ω9	0.1±0.0	0.3±0.0	.001
C20:1ω7	0.2±0.0	0.6±0.0	.000
C16:2ω4	0.7±0.0	4.4±0.0	.000
C16:3ω4	1.2±0.0	0.6±0.0	.011
C18:2ω6 (LA)	4.7±0.1	5.4±0.1	.000
C18:3ω6	0.2±0.0	1.2±0.0	.000
C18:3ω3 (LNA)	3.3±0.1	1.2±0.0	.000
C18:3ω4	6.6±0.2	1.5±0.0	.000
C18:4ω3	0.3±0.0	0.8±0.0	.000
C20:2ω6	0.2±0.0	0.6±0.0	.000
C20:3ω6	0.7±0.0	4.2±0.1	.000
C20:4ω6 (ARA)	0.5±0.1	0.7±0.0	.417
C20:3ω3	0.4±0.0	0.3±0.0	.007
C20:4ω3	1.0±0.0	0.2±0.0	.000
C20:5ω3 (EPA)	5.4±0.1	2.7±0.1	.000
C22:2ω6	0.5±0.0	2.6±0.1	.000
C22:5ω3	2.1±0.1	9.6±0.1	.000
C22:6ω3 (DHA)	22.3±0.3	6.1±0.1	.000
ΣSFA	32.4±0.3	37.3±0.2	.000
ΣMUFA	2.2±0.0	20.4±0.2	.000
ΣPUFA	50.2±0.5	42.2±0.2	.000
ΣLC-PUFA	36.3±0.4	16.1±0.1	.000
DHA/EPA	4.1±0.1	2.3±2.2	.000

## Discussion

The present study is the first to investigate the effects of domestication on the calanoid copepod *P*. *crassirostris* after 2 years of continuous culture. We observed a significant improvement in the reproductive capacity of the domesticated copepods compared to the wild copepods, with almost a 2-fold increase in egg production. In addition, the domesticated strain had a higher tolerance for different culture environments than wild copepods, as demonstrated by the low mortality rate and unaffected egg production at different culture densities.

Domestication often leads to improved adaptability to culture environments of cultured species and the enhancement of traits related to culture productivity [36]. The current results coincide with the previous statement, as the reproductive capacity of the domesticated strain exceeded that of the wild strain. [37] Studied *P*. *crassirostris* population in the same coastal area of Northern Queensland, Australia, and reported that the *in situ* mean daily egg production was very low, being only 5 eggs female^-1^ day^-1^, demonstrating that both acclimatization to optimal culture environment and domestication significantly improved the reproductive capacity of *P*. *crassirostris*. [26] reported similar results when comparing laboratory-cultured *Acartia tonsa* to wild-caught specimens, which produced only half as many eggs. However, the study compared adult females of various ages, which could have affected those results [38]. A positive correlation between female size and egg production has been described previously in copepods and indicates that larger females (prosome size) have the capacity to produce more eggs [39, 40]. However, the increase in female prosome length of the domesticated strain compared to the wild strain was only 2.7%, which could not have solely accounted for the increase in egg production. Moreover, during a selective breeding program designed to increase the reproductive capacity of *P*. *crassirostris*, [33] found that selected females had a 10% increase in prosome length with an increase in egg production of 24.5%. This report suggests that the increase in prosome length played a small role in the current experiment. The heightened egg production by the domesticated strain in the present study is likely due to improvements in oogenesis, which could be related to adaptations to the controlled culture environment (i.e., food satiated environment) [41].

Culture density is an essential parameter that is often described as the “bottleneck” in a cost-effective implementation of copepods in aquaculture [19]. The present culture density results are very promising, considering the obvious variation between both strains. The domesticated copepods sustained a higher culture density than the wild copepods. After a period of acclimatization, the wild *P*. *crassirostris* strain had a lower tolerance to the culture environment compared to the domesticated strain. This finding indicates that calanoid copepods require multiple generations of culture to reach their full potential to adjust to different culture environments. Hence, future studies on copepod culture density should reference the history of stock cultures.

Although no significant differences were found between strains when comparing faecal pellet production, the ingestion rate of the domesticated strain was higher than the wild strain. The higher ingestion rate by the domesticated copepods was achieved by adaptation to the offered food, while in nature, seasonality and food concentration is suggested to play a major role in copepod ingestion rate [42]. The change from diverse food particles with various sizes to a monospecific diet could also explain the less efficient ingestion rate of the wild strain [43].

Fatty acids are essential to the health and wellbeing of fish larvae [44]. The quantity and quality of essential fatty acids are vital for the development of fish larvae [45]. Copepods, as the natural prey of most fish larvae, provide a valuable source of essential fatty acids [15]. However, the fatty acid profile of cultured copepods can vary significantly [8, 27]. Our results showed an apparent difference between the domesticated and wild strain’s fatty acid profiles. Although the wild strain was acclimatized to the same diet for one generation, the levels of essential fatty acids were lower than the domesticated strain. The lower levels of LC-PUFAs found in the wild strain fatty acid profile could be a reflection of their assimilation capacity [43]. Nonetheless, the diet shift and stress caused by a new environment requires energy, which will rapidly change the fatty acid profile [46]. The enhanced nutritional value of domesticated copepods is a good investment, as the improvement in the fatty acid profile of domesticated copepods gives merit to long-term cultivation programs.

In conclusion, this study found that reproductive capacity and tolerance to the culture environment of the domesticated calanoid *P*. *crassirostris* has improved significantly. This improvement presents a new domain in the development of copepod culture techniques, as domestication could be a step towards the development of new copepod strains that require many generations to reach a balanced equilibrium under culture conditions. Developing copepod strains through strain selection and selective breeding could be coupled with domestication to produce a copepod strain that can be utilized by aquaculture hatcheries in the future.
